# Gender dynamics in Animal Science: progress, pitfalls, and pathways forward

**DOI:** 10.1093/af/vfaf018

**Published:** 2025-10-29

**Authors:** Paola Crepaldi, Lucia Bailoni, Anna Sandrucci

**Affiliations:** Department of Agricultural and Environmental Sciences—Production, Landscape, Agroenergy, University of Milan, Milano, Italy; Department of Comparative Biomedicine and Food Science, University of Padova, Legnaro, Italy; Department of Agricultural and Environmental Sciences—Production, Landscape, Agroenergy, University of Milan, Milano, Italy

**Keywords:** academic career, animal science, doctoral degree, gender equality, student

**Implications**

- Despite progress, gender disparities persist in higher education and academia. This paper summarizes key findings on gender imbalance in the field of Animal Science in Italy and outlines strategies for improvement.
- Although women are well represented in undergraduate and graduate programs, their numbers decline at the doctoral level and are notably low in senior academic roles.
- Gender representation varies across animal science disciplines, with greater disparities in more technically oriented areas. Women are also underrepresented in authorship and leadership roles within faculties and scientific associations.
- Barriers, such as the “glass ceiling,” “leaky pipeline,” and “sticky floors”, continue to limit women’s career progression.
- Addressing these issues requires comprehensive reforms in evaluation criteria, mentorship programs, work-life balance policies, and institutional accountability. Regular monitoring and publishing of gender-disaggregated data is crucial for tracking progress. True change will require joint efforts from universities, research institutions, and scientific associations.

## Introduction

Gender equality is one of the 17 core objectives of the Sustainable Development Goals (SDGs) outlined in the 2030 Agenda for Sustainable Development, adopted in 2015 by all 193 United Nations member states. As stated in SDG 5—*Achieve gender equality and empower all women and girls*—“Gender equality is not only a fundamental human right but a necessary foundation for a peaceful, prosperous and sustainable world.”

**Figure 1.**
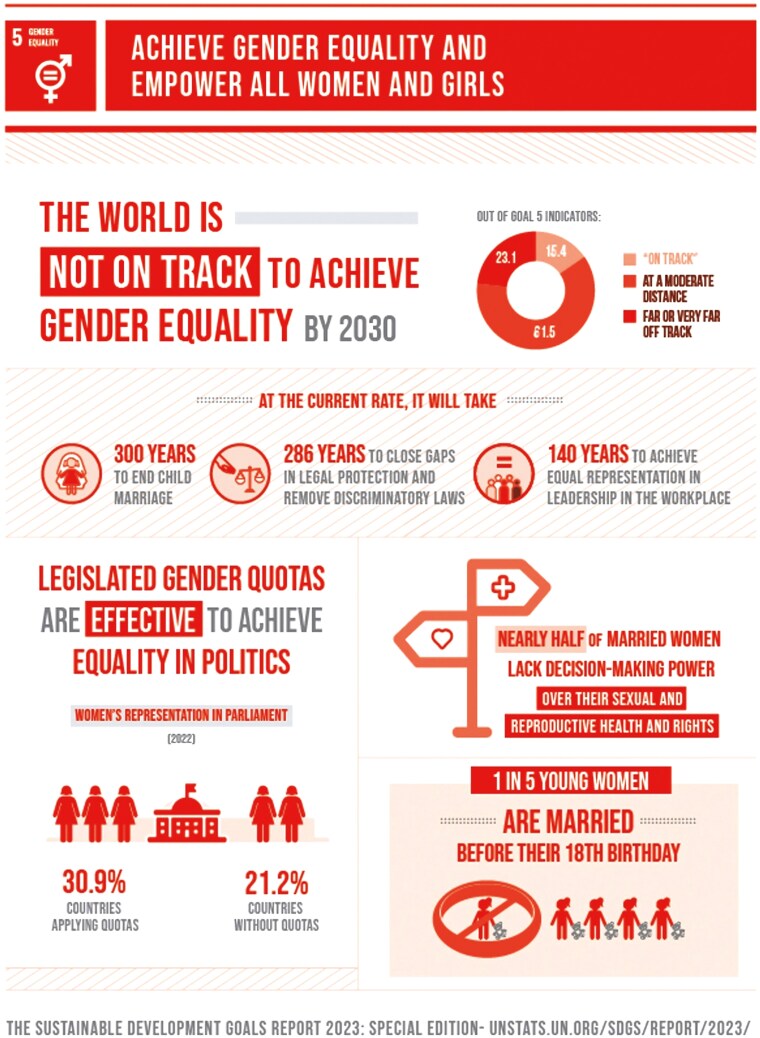
Progress toward SDG 5—gender equality. Source: United Nations (2023).

According to the United Nations SDG 2023 Report, the world is unlikely to meet the target of gender equality by 2030 (Figure 1; United Nations, 2023). Significant delays remain in key areas such as ending child marriage, ensuring legal protection, increasing women’s political representation, and promoting female leadership. Achieving this goal requires proactive efforts to recognize and strengthen women’s skills and to ensure equal opportunities for leadership and participation in decision-making processes at all levels.

Gender disparities in higher education and research persist across Europe, particularly in the fields of Science, Technology, Engineering, and Mathematics (STEM). Although women represent an increasing proportion of students, doctoral candidates, and early-career researchers, their numbers decline markedly in senior academic and leadership roles, a pattern well documented in the *She Figures* report (European Commission, 2025).

Women are more likely than men to complete Bachelor’s degrees, including in STEM fields. This pattern is evident in Italy as well, both in general and within the fields of agricultural and veterinary sciences. Although not always explicitly included in narrow definitions of STEM (Science, Technology, Engineering, Mathematics) or STEMM (which adds Medicine), Animal Sciences and Animal Production rely heavily on scientific and technological approaches. Applying tools and methods from biology, veterinary medicine, data science, and engineering, they fall within a broader and inclusive understanding of STEMM.

While women outnumber men among Master’s graduates, they are less likely to progress to Doctoral-level studies (8% vs. 11%), including in STEM and agricultural/veterinary sciences (European Commission, 2025).

At the EU level, gender balance among Doctoral graduates has nearly been achieved (48% women), but disparities remain across disciplines: women account for over 60% of Doctoral graduates in Education, while their share in STEM has declined since 2018, reversing earlier progress toward parity. In Italy, the proportion of women among all Doctoral graduates fell by over three percentage points between 2013 and 2024, an unfavorable trend that raises concern. However, women remain well represented in agricultural and veterinary sciences, making up about 58% of Doctoral graduates both in Italy and across the EU (European Commission, 2025).

Across disciplines, gender balance has nearly been achieved among students at all levels (Bachelor’s, Master’s, and Doctoral) as well as among grade C (entry-level) and grade B (mid-senior) academic staff. However, women hold only 30% of top academic positions (grade A), even in fields where they are otherwise well represented. Since 2019, progress has been modest, with increases of just 1 to 2 percentage points across academic grades. Women remain underrepresented in leadership roles: they make up only 26% of heads of institutions, while gender balance is closer to being achieved among board members and board leaders (both at 39%) (European Commission, 2024).

The “She Figures 2024” report shows that, in 13 out of 36 countries analyzed, at least half of the research organizations implement gender equality measures. However, despite formal commitments and ongoing initiatives, persistent gender disparities remain, particularly in leadership positions, authorship roles, and career progression.

The authors of this paper are promoters of the ASPA Working Group on gender equality, which aims to promote awareness, monitoring, and debate to overcome gender segregation in the association. The article examines gender disparities within the field of Animal Science in Italy, building on data from a previous study (Sandrucci et al., 2025) and integrating new findings. It aims to offer concrete recommendations to promote gender equality in education and research in this discipline that can be extended to other disciplines as well.

## Key Observations

### Higher education

In Italy, Master’s and single-cycle degree programs in Animal Sciences fall into three main degree classes: Animal Production Sciences, Agricultural Sciences, and Veterinary Medicine. In 2023, there were 1,889 graduates in these programs, 49.7% of whom were women. However, gender distribution varies considerably across the three tracks (Almalaurea, 2025a).

Over the past decade, between 2013 and 2023, Master’s degree graduates in Animal Production Sciences increased by 65%, from 161 to 267, with the proportion of women rising by 5.8 percentage points from 60.9% to 66.7%. The number of Master’s degree graduates in Agricultural Sciences has more than doubled over the same period, from 446 to 984, with women increasing modestly by 2.7 percentage points, from 28.3% to 31%. It should be noted that this category includes all agronomists working in animal husbandry but also in other sectors of agricultural science. In contrast, the number of graduates in Veterinary Medicine decreased by 20.5% in the same decade, from 803 to 638, due to a national policy decision. The percentage of women, however, increased by about 5 percentage points, from 66.4% to 71.5%.

In summary, in 2023, women accounted for 66.6% of animal production graduates, 31% of agronomists, and 71.5% of veterinarians.

In both 2013 and 2023, the majority of women graduating from Master’s and single-cycle degree programs in Animal Sciences (77% in 2023) had attended academic-track secondary schools (classical studies, scientific studies, foreign languages, human science, art, music, and dance), a share over 20 percentage points higher than that of men, who frequently came from technical institutes (43% in 2023). Women had also achieved higher high school diploma scores (on a 100-point scale) prior to enrolling at university, with the most notable difference (about 5 points) observed in Veterinary Medicine.

This academic advantage persisted throughout university studies. Female graduates consistently obtained higher final degree marks, especially in Veterinary Medicine, where the difference reached about 2 points on average in 2023. In addition, the proportion of students graduating on time was higher for women, particularly in Animal Production Science programs, where the gap in 2023 exceeded 10 percentage points in their favor.

Participation in study abroad programs declined over the decade across all Animal Science tracks but female students participated slightly more than their male counterparts.

A more persistent gender gap emerged in work experience during university, especially in activities directly related to the field of study. In 2023, the overall proportion of students reporting work experience ranged between 60% and 70% across all three study tracks. However, fewer women than men reported having relevant work experience in Animal Production Science and Agricultural Sciences, while the opposite was true in Veterinary Medicine. Women were also more likely to gain work experience outside their field of study, particularly in Agricultural and Animal Production Sciences.

In summary, over the past decade, women enrolled in Italian Master’s and single-cycle degree programs related to Animal Sciences (Animal Production Sciences, Agricultural Sciences, and Veterinary Medicine) have consistently outperformed men in terms of academic achievement, with higher grades and on-time graduation rates. However, they often acquire less practical, field-specific experience during their studies, which may place them at a disadvantage when entering the workforce despite their academic excellence.

Several factors may contribute to the lower rate of field-specific work experience among female students. Gender-based social expectations and self-perceptions can discourage female students from pursuing roles in traditionally male-dominated sectors such as agriculture and livestock production. Rural spaces are often perceived as masculine domains, and women may face skepticism, not only about their competence but also about the legitimacy of their presence. Research has shown that women in farming can encounter resistance from male colleagues, including withholding of information and reluctance to acknowledge their professional legitimacy (Smyth et al., 2018). Additionally, women may prioritize academic achievement over practical experience, consciously or unconsciously compensating for anticipated disadvantages in the labor market. While this focus leads to strong academic records, it may also limit opportunities to build the practical skills and professional networks that employers in the sector value.

### Doctoral degrees

In Italy, earning a doctoral degree is a key step toward an academic career. Considering all disciplinary areas, female doctoral candidates in Italy in 2023 constituted slightly less than half (49.4%) of the total (MUR USTAT, 2025). This percentage is significantly lower compared to that observed for second-level graduates (Master’s degree) from the same source (58.6%), confirming a lower propensity for women to pursue research in academia. In Agricultural and Veterinary Sciences, however, women accounted for 57.1% of PhD graduates, a figure that has remained stable over the past decade. AlmaLaurea (2025b) confirms this trend: in the Life Sciences area, the share of women drops from 60.2% among Master’s graduates to 48.5% at the doctoral level.

In the decision to pursue a doctorate, as emerged from a recent survey by Carriero et al. (2024), individual motivations can be significantly influenced by gender, taking into account the challenges for women entering a highly competitive environment, their lower propensity for risk-taking, and, above all, the difficulties in reconciling family and work commitments. Even outside academia, female Doctoral graduates are disadvantaged compared to males: one year after completing the doctorate, they face more difficulties entering the job market, earn less, and are more likely to work part-time (AlmaLaurea, 2025b). The system fails to fully capitalize on the academic excellence of female graduates, reducing the return on public and private educational investment. With regards to the doctorate, it is difficult to isolate data relating exclusively to Animal Science PhDs from the Life Sciences area (213 PhD courses in Italy), since the doctorate courses are usually multidisciplinary, typically encompassing Food, Environmental, Health, and Veterinary Sciences.

### Academic career

The academic positions and scientific sectors in the Italian university system were collected by consulting the archive of the Italian Ministry of University (CINECA, 2025). In Italy, Scientific Disciplinary Sectors (SDSs) are used to classify academic disciplines and organize research activities. Each SDS corresponds to a specific field, guiding researchers’ specialization and serving as a framework for evaluating scientific productivity through qualification procedures based on sector-specific thresholds. In the field of animal sciences, the SDSs related to animal husbandry include:

- Animal breeding and genetics (AGRI-09/A);
- Animal nutrition and feeding (AGRI-09/B);
- Husbandry techniques (AGRI-09/C);
- Short-cycle animals (fish, poultry, rabbit, laboratory animals) (AGRI-09/D).

**Figure 2.**
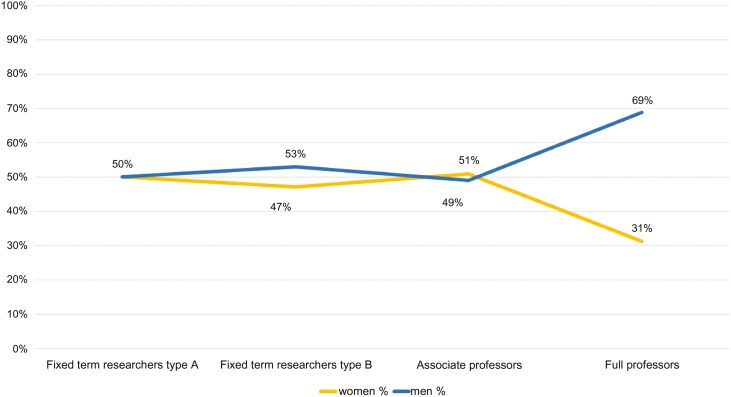
Gender distribution within Italian academic staff in the Scientific Disciplinary Sectors of Animal Science in the year 2025 by position (excluding tenured researchers).

The percentages shown in Figure 2 represent the proportion of women and men in the four Animal Science SDSs at the national level within the Italian university system. Tenured university researchers are excluded, as this position is being phased out and is no longer renewed.

Overall, women make up 44% of academic staff in these sectors and are well represented among junior researchers. However, they remain underrepresented among full professors (31%). Gender distribution also varies by discipline: in more technical areas like Animal Genetics (AGRI-09/A), women account for only 35% of academics, and just 25% of full professors. In contrast, disciplines such as Short-Cycle Animals (AGRI-09/D) are close to gender parity or show a female majority.

Figure 2 illustrates the typical “scissor-like” trend in the male-to-female ratio across academic career levels: women are the majority in lower-level positions but become a minority as rank increases. This reflects vertical segregation, accompanied by horizontal segregation across disciplinary fields. The pattern closely resembles that observed in a previous study on gender equality within the Italian Association of Animal Science and Production (Sandrucci et al., 2025).

This trend is also confirmed, with some variations, by national data on academic staff in 2020 (Gaiaschi, 2022), and at the European level by “She Figures 2024” report (European Commission, 2024).

In 2023, across the entire Italian university system, full professors accounted for 31% of the total male academic staff (including full and associate professors as well as fixed-term researchers), compared to 18% among women (MUR USTAT, 2025).

**Figure 3.**
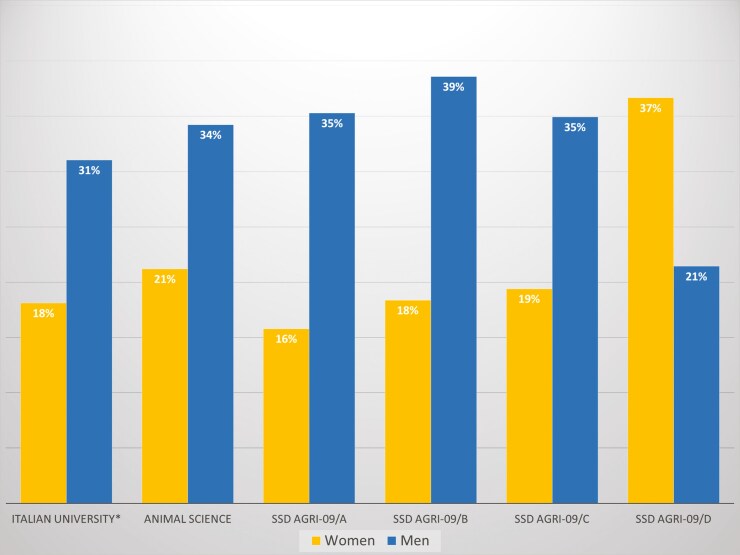
Proportion (%) of grade A staff among all academic staff by sex, 2025 (CINECA, 2025). *For Italian University data are referred to 2023 (MUR USTAT, 2025).

In the field of Animal Sciences, according to CINECA data (2025), these proportions were 34% for men and 21% for women. In the specific subfield of Genetics (AGRI-09/A), the figures were 35% for men and 16% for women (Figure 3).

Regarding scientific publications, an analysis of authorship trends in the official journal of the Italian Association of Animal Science and Production, the “Italian Journal of Animal Science”, revealed further gender disparities (Sandrucci et al., 2025). Women are significantly underrepresented in senior authorship positions, particularly as last authors, a role typically associated with project leadership or senior academic contribution.

A similar pattern has been observed in veterinary research: according to Giuffrida et al. (2019), among 1,043 articles published in 2015, women were first authors in 60.0% of cases but accounted for only 38.3% of senior authorship. Comparable findings have also emerged in the field of animal cognitive sciences (Gavriilidi and Van Damme, 2023).

At the European level, data confirm that women remain significantly underrepresented as authors compared to men, with the gender gap becoming more pronounced at higher levels of academic seniority (European Commission, 2024).

Bibliometric data offer additional insight. Gender differences in h-index values (a metric that reflects both the number of a researcher’s publications and how often they are cited) and publication counts are statistically significant but largely reflect disparities in academic position (Sandrucci et al., 2025). In other words, research productivity is not determined by gender per se, but by access to positions that offer greater opportunities, resources, and collaborative networks.

The literature widely acknowledges that male researchers, on average, have higher publication outputs than their female counterparts. This is largely because women are overrepresented in lower academic ranks and temporary positions, which are often associated with heavier teaching loads, limited access to funding, fewer career prospects, and restricted resources for research. As a result, there is a well-established positive relationship between job level and research productivity. Gender thus influences academic rank, research roles, and network positioning: female researchers are less likely to occupy senior or leading roles, which negatively affects their research and publishing performance, slows down their career progression, and further reinforces their underrepresentation at the top, a self-perpetuating dynamic. As Van den Besselaar and Sandström (2017) argue, this vicious cycle may help explain the persistence of gender disparities in academia.

The imbalance also extends to governance and visibility. Although the proportion of women chairing sessions at the Italian Association of Animal Science and Production congresses has increased over time, invited speakers remain predominantly male. Similar patterns have been reported in other scientific disciplines (see, among others, Débarre et al., 2018). Leadership within the Italian Association of Animal Science and Production continues to be male-dominated, reflecting deeper structural inequalities in access to influence and decision-making (Sandrucci et al., 2025). A similar trend is evident in the veterinary field: despite the growing feminization of the profession and the increasing number of female students and professionals, a global analysis of 720 veterinary institutions revealed that only 34.6% of executive leadership positions were held by women, and just 25.5% of top roles (deans) were occupied by women (Vezeau et al., 2024).

### Barriers to gender equality in academia

A substantial body of literature highlights numerous factors contributing to gender inequalities and underrepresentation of women in leadership roles in academia. These include conflicting life, family, and work priorities; lack of self-efficacy and confidence; feelings of marginalization and isolation; insufficient mentoring; limited access to professional networks; discriminatory environments; cultural conditioning; and unconscious bias. One of the most persistent structural obstacles is the so-called glass ceiling, an invisible barrier that prevents women (and minority groups) from advancing beyond a certain level in the professional hierarchy. Well documented in academia (Brown et al., 2020) , the glass ceiling is reinforced by implicit bias, institutional cultures that undervalue women’s contributions, and exclusionary informal networks (Cannito et al., 2023).

A second well-documented challenge is the “leaky pipeline,” a metaphor used to describe the gradual loss of women (and other underrepresented groups) at key transition points in educational and career pathways as they move toward higher and more stable positions (Falco et al., 2023). This phenomenon is particularly evident between the doctoral and postdoctoral stages, where women often face the combined pressures of family responsibilities, job insecurity, and limited mentorship, especially in STEM and technical disciplines (Avargil et al., 2023).

Furthermore, the concept of “sticky floors” refers to the difficulty many women face in advancing beyond entry-level or temporary positions (Falco et al., 2023). Often confined to roles with limited long-term prospects, women may struggle to build the same professional capital as their male peers due to insufficient support, mentoring, funding opportunities, and disproportionate family responsibilities.

Evaluation systems based on traditional, linear academic careers exacerbate these inequalities. Career interruptions, more common among women, are penalized in systems that reward continuity and uninterrupted productivity (Eagly, 2020).

Gaiaschi (2022) argued that a double standard persists in academia: women are evaluated more harshly than men under identical conditions, particularly in promotions, making career progression slower and more difficult. Her work explores the link between the feminization of certain fields and the persistence of gender inequality in academia, even compared to the broader labor market. Despite the growing presence of women, gender asymmetries continue to reproduce themselves. Based on years of research, Gaiaschi highlights key objections she encountered, especially regarding the shift from STEM to STEMM. While traditional STEM fields have been male-dominated, the inclusion of Medicine (and by extension, applied life sciences such as biology, veterinary medicine, and agricultural sciences) has led to female majorities in several areas. Yet, inequalities remain, particularly in advancement opportunities, even in these “feminized” disciplines. She presented two key arguments that slow down the implementation of concrete actions:

- invisibility of inequality: gender disparities are often hidden and therefore dismissed. Even when women succeed, the extra challenges they face are frequently ignored.
- the demographic (or time) argument: some claim existing disparities are generational and will naturally disappear over time, a view known as the *critical mass illusion*. This perspective undermines the urgency for structural change, despite evidence showing that barriers persist even where numerical gender balance has been achieved.

In addition, Gaiaschi identifies some of the most common forms of resistance, including denial of inequality, which involves citing the success of a few prominent women as evidence that gender inequality no longer exists, while ignoring broader structural disadvantages. This is known as the *token paradox*.

Another common form of resistance is the so-called conservative justification, which consists of attributing gender differences to supposed innate preferences (e.g., choosing education over STEM fields) or life choices (e.g., prioritizing family over career).

## Recommendations

The lack of gender equity in academia has significant implications for the overall fairness and innovation capacity of the academic system (Bührer et al., 2020). Diversity enhances creativity, problem-solving, and productivity, and benefits all three missions of academia: research, teaching, and outreach. Overcoming gender disparities is therefore crucial to advancing research quality and impact.

Finding shared and effective solutions is not easy, given the complexity of underlying causes and, as Gaiaschi (2022) notes, the persistence of resistance and the tendency to underestimate the problem.

To promote gender equality in academia, including in Animal Science, it is essential to ensure transparent and inclusive selection processes, with balanced representation in recruitment, tenure, and promotion committees. Many institutions now require training on unconscious bias and equity policies for committee members. Recruitment panels are increasingly expected to include bias reduction training to support diverse hiring (Carr et al., 2017).

This training should also be extended to department heads, deans, university and departmental board members, as well as members of national scientific qualification committees and panels evaluating research funding proposals.

Evaluation systems must be reformed to recognize diverse career trajectories. Inclusive metrics should acknowledge the value of teaching, outreach, and interdisciplinary collaboration, moving beyond narrow bibliometric indicators.

Institutional policies should support career progression through structured mentorship and sponsorship programs tailored for early-career women researchers. Mentoring programs, based on the sharing of experience and networks by senior professionals, can help address the barriers associated with the leaky pipeline and sticky floors (Powell S., 2018) .

Monitoring and publishing gender-disaggregated data, such as the percentage of women applying for and obtaining academic positions, research funding, authorship roles, leadership positions, memberships in scientific associations, and participation in scientific events, is essential to ensure transparency and accountability. To strengthen institutional responsibility, the adoption of Gender Equality Plans (GEPs), defined as a structured set of measures and actions aimed at promoting gender equity, should be made mandatory for access to all competitive research funding. Moreover, institutions should undergo regular evaluations to assess the effectiveness of their gender policies and the progress made toward achieving equitable outcomes.

Affirmative action and quotas are widely used methods aimed at creating a critical mass of underrepresented groups in order to end discrimination in academia (Powell, 2018). Gender-balanced representation in scientific committees, editorial boards, scientific congresses, and public events should be a norm rather than an exception. Including gender ratios in event programs and requiring diversity in organizing committees can raise awareness and stimulate proactive change.

At the same time, cultivating inclusive institutional cultures that promote work-life balance and support caregiving responsibilities is essential for retaining female talent in academia.

## Conclusion

Gender inequality in animal science and in academia remains a critical issue that undermines scientific excellence and limits the contribution to social equity and innovation. This article has examined persistent gender disparities in the field of Animal Science in Italy, providing an overview of educational trajectories, academic careers, authorship patterns, and institutional dynamics. Despite the steady increase in the number of women graduating in Animal Sciences, often with higher academic performance than their male peers, structural inequalities continue to limit their access to practical experience, doctoral studies, senior academic positions, and leadership roles.

The analysis confirms that gender imbalance follows a typical “scissor-like” pattern: women are well represented at early-career stages, but their presence declines sharply at higher levels. This vertical segregation is compounded by horizontal segregation, with women still underrepresented in more technical and male-dominated subfields. These disparities extend beyond academic hierarchies, affecting authorship, research visibility, and access to institutional leadership.

Barriers such as the glass ceiling, leaky pipeline, and sticky floors persist, driven by a complex interplay of structural limitations, implicit biases, institutional cultures, and gender stereotypes. Even in fields with a strong female presence, such as Veterinary Medicine, women face slower career advancement, reduced access to leadership roles, and continue to be evaluated through outdated systems that fail to recognize diverse academic trajectories. Gender equality will not be achieved spontaneously over time, but it requires targeted, concrete action.

This article has outlined a set of strategic recommendations to promote gender equity: reforming selection and evaluation procedures, implementing mentorship programs, adopting GEPs, and fostering inclusive academic environments. While some of these measures are already in place in certain institutions, significant efforts are still needed to ensure their full implementation and effectiveness.

Achieving meaningful and lasting change will require coordinated action across the academic ecosystem, involving universities, research institutions, funding agencies, and scientific associations. Only through sustained commitment and collaboration can the field of Animal Science become more equitable, innovative, and resilient in the face of future challenges.

## About the Authors

**Paola Crepaldi** is Associate Professor of Animal Breeding and Genetics at the University of Milan, Department of Agricultural and Environmental Sciences (DISAA). She holds a degree in Agricultural Science and a PhD in Animal Nutrition and Technologies, and has been nationally qualified as a Full Professor since 2017. She teaches Animal Breeding, Data Analysis, and Biostatistics in master’s programs focused on agricultural sustainability and biotechnology. Her research focuses on the genomic biodiversity of farm and companion animals, especially population structure, selection signatures, and climate change impact. She has authored numerous peer-reviewed publications, and she is actively involved in the Italian and European Association of Animal Science and Production. She is also actively involved in the study and promotion of the role of women in science. She is serving as coordinator of the Italian Goat Genome Consortium, and President of the Central Technical Commission for the herd books of Italian goat breeds.

**Lucia Bailoni** is a Full Professor in Animal Nutrition at the University of Padua (Italy), Department of Comparative Medicine and Food Science. She graduated with honors in Agricultural Sciences and earned a PhD in Animal Sciences. Prof. Bailoni teaches Animal Nutrition at the School of Agriculture and Veterinary Medicine in the Agripolis Campus (Legnaro, PD, Italy). Her research focuses on improving the nutritional value of food of animal origin (dairy milk), reducing the environmental impact of livestock, and enhancing dairy farm sustainability. She is responsible for the Fermentation Lab for in vitro ruminal metabolism studies. She served as Director of the Department of Comparative Biomedicine and Food Science (2015–2019) and is currently its Third Mission Representative. Nationally, she is Vice-President of the Italian Association for Science and Animal Production (ASPA).

**Corresponding author**: lucia.bailoni@unipd.it

**Anna Sandrucci** is a Full Professor of Animal Husbandry at the University of Milan. Her research focuses on dairy cattle, the environmental impact of animal production, Precision Livestock Farming, animal welfare, and the quality of food of animal origin. She has authored or coauthored approximately 200 scientific and technical publications, including 64 peer-reviewed articles indexed in Scopus. She has contributed as author and editor to a number of books on animal science and related topics. She is a member of the Accademia dei Georgofili in Florence and an ordinary member of the Italian Association for Animal Science and Production (ASPA). She serves on the executive boards of the Società Agraria di Lombardia and the Museum of the History of Agriculture. She is also: member of the ASPA Scientific Committee on Precision Livestock Farming and of the ASPA Working Group on Gender Equality; Member of the Working Group of UNASA (National Union of Academies for Applied Sciences in Agriculture, Food Security and Environmental Protection); UNIMI representative on the Scientific and Technical Committee of Farming Future, the National Agritech Technology Transfer Centre Founder and member of the AGRIFOOD-LCA Lab, a University Research Laboratory on Life Cycle Assessment in Agri-Food Chains.
